# Comparing In Vitro Faecal Fermentation Methods as Surrogates for Phage Therapy Application

**DOI:** 10.3390/v14122632

**Published:** 2022-11-25

**Authors:** Norbert Ács, Ross Holohan, Laura J. Dunne, Adrian R. Fernandes, Adam G. Clooney, Lorraine A. Draper, R. Paul Ross, Colin Hill

**Affiliations:** 1 APC Microbiome Ireland, University College Cork, T12 YT20 Cork, Ireland; 2 School of Microbiology, University College Cork, T12 K8AF Cork, Ireland

**Keywords:** bacteriophage, phageome, bacteriome, human gut microbiome, faecal fermentation

## Abstract

The human microbiome and its importance in health and disease have been the subject of numerous research articles. Most microbes reside in the digestive tract, with up to 10^12^ cells per gram of faecal material found in the colon. In terms of gene number, it has been estimated that the gut microbiome harbours >100 times more genes than the human genome. Several human intestinal diseases are strongly associated with disruptions in gut microbiome composition. Less studied components of the gut microbiome are the bacterial viruses called bacteriophages that may be present in numbers equal to or greater than the prokaryotes. Their potential to lyse their bacterial hosts, or to act as agents of horizontal gene transfer makes them important research targets. In this study in vitro faecal fermentation systems were developed and compared for their ability to act as surrogates for the human colon. Changes in bacterial and viral composition occurred after introducing a high-titre single phage preparation both with and without a known bacterial host during the 24 h-long fermentation. We also show that during this timeframe 50 mL plastic tubes can provide data similar to that generated in a sophisticated faecal fermenter system. This knowledge can guide us to a better understanding of the short-term impact of bacteriophage transplants on the bacteriomes and viromes of human recipients.

## 1. Introduction

The importance of the human microbiome has been the focus of research for more than a decade. Bacteria residing on the skin, in the oral cavity or the vagina have been heavily investigated and as a consequence, numerous species have been implicated as key players in both health and disease [1,2,3,4,5,6]. The gut microbiome has been linked to neuronal development, obesity, cancer, diabetes, and various enteric diseases (e.g., inflammatory bowel disease) [7,8,9,10,11,12]. Recent findings suggest a connection between our gut microbiome and depression, neurodegenerative syndromes or even autism [13,14,15].

Bacteriophages (phages; viruses that prey on bacteria) are probably the most abundant biological entities on earth. Despite their small size and relatively limited genome content, they can play a crucial role in shaping the bacterial population of many environmental habitats including soil, seawater and multiple niches associated with animals or humans. Phages can also act as agents of horizontal gene transfer [16]. Phages have been used to treat bacterial infections in humans for close to 100 years [17,18], but since the development of antibiotics, the use of phages against infectious diseases has not been widely practiced. However, the overuse of antibiotics in recent decades has led to the development of multidrug-resistant human pathogens, imposing a major burden on healthcare systems worldwide. This has fuelled a renewed interest in using virulent phages as alternatives to antibiotics. Phage therapy is currently gaining momentum as ingestible or locally administered forms with numerous clinical trials already complete, and more underway [19].

Our understanding of the viral component of the gut microbiome (the phageome) in humans remains limited. A number of studies have examined the viral fraction in healthy populations [20,21] and different disease states compared to healthy controls [22,23,24,25,26]. Due to a lack of extensive referenced phage sequence databases the majority of the human gut virome is comprised of unknown members, hence the term “viral dark matter” is frequently used. Nevertheless, several possible applications involving gut phages are already available. For example, the recently identified CrAssphage–the most abundant member of the human gut virome–was proposed as a marker for environmental faecal contamination [27,28]. Another intensely investigated clinical practice is faecal material transplantation (FMT) which is often used to treat recurrent *Clostridioides difficile* infections [29,30]. During FMT, phages may have a limited effect, since numerous washing steps can deplete the non-bacterial fraction; however, one study specifically used faecal filtrate (FFT) and saw a similar treatment effect without live bacteria. This suggests that phages could play a role in restoring a healthy gut bacterial population [31]. We recently published an FMT follow-up study where the fate of phages originating from three donors was followed for 12 months. The 14 FMT-treated patients showed evidence of extensive engraftment of phages from the donor phageome [32].

In vivo human and animal intervention studies require extensive background data for ethical and animal welfare reasons. This is usually generated by in vitro pilot experiments to collect preliminary data about the efficacy and safety of phage use. Faecal fermentations are often used as a proxy for the human colon but generally accepted guidelines for faecal fermenter setup are currently lacking. Different research groups have reported using either custom-made or commercially available sophisticated equipment [33,34,35]. Both involve a relatively high purchase cost; a long time to set up, maintain and run experiments; are labour-intensive and produce a lot of biohazardous waste that needs to be safely disposed of. Others used a simpler batch-type fermenter setup. However, none of these studies investigated how phages behave during these conditions [36,37,38].

Commonly applied model systems cannot perfectly mimic the complex human gut system in both physical and chemical aspects. Nevertheless, they serve as a good proxy and information gathered using these model systems can advance our understanding of the interplay between phages and their hosts in these complex environments [39]. In this study, we performed 24 h-long in vivo faecal fermentations using faecal tubes and faecal vessels run in parallel. The same human faecal standard inoculum (FSI) and growth medium as starting material. Applying either a commercially available sophisticated two-vessel system (referred to as vessels) or a simpler setup adopting widely used 50 mL plastic centrifuge tubes (referred to as tubes). We aimed to investigate the short-term effect of a single phage on a gut bacteriome and virome, allowing us to perform a comprehensive comparison of both approaches within this period. This timeframe was selected to reflect the typical colonic transit time. Phage interventions were carried out with ΦAPCEc01, a human gut-associated lytic phage that was isolated in our laboratory from a faecal sample of an anonymous donor and which is capable of infecting a range of *Escherichia coli* strains [40]. ΦAPCEc01 was introduced at high titre either on its own or in combination with a known bacterial host (*E. coli* APC106). Using 16S rRNA amplicon sequencing to estimate bacterial abundances and ecological community measures such as species richness and evenness (α and β diversities, respectively), it was possible to critically compare the impact of both setups on the bacterial population, while WGS of the viral enriched part shed light on the virome.

Here, we report the impact of fermenter setup and different treatment conditions on bacterial and viral populations occurred during the 24 h timespan. Our results can help to assess the safety and efficacy of human intestinal phage therapy in a more economical way.

## 2. Materials and Methods

### 2.1. Bacterial Strain, Phage and Inoculum Used

*Escherichia coli* APC106 originated from the APC Microbiome Ireland’s culture collection, while ΦAPCEc01 was isolated in our laboratory previously [40]. Faecal samples were donated by anonymous donors with ethical approval. The faecal standard inoculum was produced according to M. O’Donnell et al. [41]. Total phageome was isolated parallel with FSI generation using the supernatant that is normally a by-product of the preparation. After decanting, the supernatant was further centrifuged at higher speed (15,000 RPM) and filtered through sterile 0.45 µm syringe filters (Sarstedt AG, Nümbrecht, Germany). Where concentration of the viral fraction was needed, a two-hour-long ultracentrifuge step was introduced at 40,000 RPM followed by resuspension of the pellet in an appropriate amount (500–1000 µL) of SM buffer (100 mM NaCl, 8 mM MgSO_4_ × 7H_2_O, 50 mM Tris-Cl pH 7.5 in 1L H_2_O).

### 2.2. Two-Vessel Fermentation System

The MiniBio reactor system from Applikon Biotechnology^®^ (Delft, The Netherlands) was used for batch fermentation. These reactors were designed for biological fermentations, hence temperature, pH, stirring and anaerobic conditions were continuously measured and/or maintained. Fastidious anaerobe broth (Lab M, Lancashire, UK) was used as the growth medium. All fermentations were operated for up to 24 h with sampling after 0 h–T0, 2 h–T1, 4 h–T2, 6 h–T3, 24 h–T4 if not stated otherwise. FSI was inoculated into each vessel filling 10% of the reactor volume. The bacterium (where present) was inoculated using 1% overnight (O/N) culture (colony forming unit–CFU 1–5 × 10^8^), also 1% of high-titre (plaque forming units–PFU 1–5 × 10^9^) phage was added where indicated. During the course of the experiment, liquid samples were withdrawn at regular time points. Approximately 10 mL fermenter effluent was placed in a 15 mL sterile centrifuge tube (Sarstedt AG, Nümbrecht, Germany) and used for further downstream analysis (bacterial and viral DNA extraction, plating on selective media, and double layer agar spot assays).

### 2.3. 50 mL Plastic Tube Fermentation System

Parallel to the faecal fermentation system a more simplified version of the same set of experiments was carried out. Parameters were as described in the previous section with appropriately decreased liquid volume. This allowed identical physicochemical parameters except for pH control and absence of stirring. After setting up the fermentation in 50 mL sterile screw cap tubes (Sarstedt AG, Nümbrecht, Germany), the content was mixed vigorously and placed into an anaerobic jar with the caps loosened. Oxoid anaerogen gas packs (Thermo Fisher Scientific, Waltham, MA, USA) were placed alongside the tubes to generate anaerobic conditions.

### 2.4. Sample Preparation Plating and Spot Assay

Liquid samples were further processed as described. 100 µL fermenter liquid was separated and serially diluted for plating. Chromocult (Merck KGaA, Darmstadt, Germany) selective medium was used to verify the presence of colony forming units (CFU) corresponding to the inoculated bacterium. The medium is selective for coliforms and employs an enzymatic reaction to colour different bacterial colonies. Native *E. coli* strains were displayed in dark purple, while the inoculated *E. coli* APC106 showed blue colour. The remaining liquid sample was centrifuged at 4700 RPM for 15 min to separate the solid fraction that was used for total bacterial DNA isolation. Supernatant was further filtered with sterile 0.45 µm syringe filters. 100 µL was set aside for spot assay, while the remaining volume was used for viral DNA extraction. Double-layer agar spot assay was performed using 1.5% LB (Luria-Bertani) base agar where 400 µL of O/N APC106 bacterium culture was mixed with 4 mL top agar (0.2% LB agarose) and left to solidify for 30 min. Subsequently, 10 µL of each centrifuged and filtered supernatant sample was spotted on the top agar and left to dry for 10 min. Plates were incubated O/N at 37 °C. Samples showing lytic activity (plaque formation) against the host bacterium strain were further diluted to assess plaque forming units (PFU).

### 2.5. DNA Isolation

The Qiagen Stool Mini kit (Qiagen, Hilden, Germany) was used to isolate total bacterial genomic DNA from the pelleted fermenter liquid following the manufacturer’s recommendations. The only modification was the decreased volume of the elution buffer to recover nucleic acid from the column (40 µL instead of 100 µL was used). For isolating the viral DNA, an in-house method was employed [42]. The only modification was the decreased elution buffer volume (40 µL).

### 2.6. 16S Amplicon Sequencing

Amplification of partial 16S rRNA gene was performed according to Shkoporov et al. using the Illumina MiSeq platform [42].

### 2.7. Viral DNA Sequencing

Accel-NGS 1S Plus DNA Library Kit for Illumina (Swift Biosciences, Ann Arbor, MI, USA) was employed to prepare sequencing libraries from isolated DNA of the viral enriched samples. This includes a shearing step at the beginning to yield the 2–300 bp sized DNA fragments using the Covaris M220 Focused-ultrasonicator (Covaris INC., Woburn, MA, USA). All subsequent reactions were carried out according to the manufacturer’s recommendations. Agilent 2100 Bioanalyzer (Agilent Technologies, Santa Clara, CA, USA) was used to verify library DNA quality. Sequencing was performed on the Illumina HiSeq platform by GATC Biotech AG (Konstanz, Germany).

### 2.8. Bioinformatics and Statistical Analysis

Raw sequencing reads were downloaded from the company’s website and processed according to our laboratory pipeline for 16S samples. This was comprised of read quality assessment on the raw reads using FastQC before and after quality filtering using Trimmomatic [43] under the following parameters; HEADCROP:15 CROP:235 SLIDINGWINDOW:4:20 MINLEN:30. The trimmed 16S reads were then further filtered (truncLen = 230, maxEE = 1.4, truncQ = 11) and processed using DADA2 [44] v1.10.1. Chimeras from the dataset were removed de novo by reference-based chimera removal using UCHIME (v4.2) [45] against the ChimeraSlayer Gold database. Resulting non-chimeric reads were sorted by length, with all having a minimum length of 200 bp and a maximum of 260 bp retained. Classification of retained 16S reads was achieved using mothur [46] (v1.38.0, bootstrap ≥ 80), while SPINGO [47] (v1.3, bootstrap ≥ 0.8, similarity ≥ 0.5) was used for species-level classification using the RDP v11.4 database as a reference.

Similarly, raw reads from the whole genome shotgun sequencing (WGS) were quality assessed using FASTQC and filtered utilizing Trimmomatic [43] with the following parameters; SLIDINGWINDOW: 4:20, MINLEN: 60 HEADCROP 15; CROP 225. Human reads were removed using Kraken [48] (v.0.10.5) and version 38 of the human genome. For contig assembly, metaSPAdes [49]) was chosen with default parameters. Removal of bacterial contamination was achieved by an extensive set of inclusion criteria favouring the selection of viral sequences only. Briefly, contigs were required to fulfil one of the following criteria; (1) Categories 1–6 from VirSorter (run with default parameters) and Refseqdb (−db 1) [50] positive, (2) circular genome organization, (3) contains a minimum of 2 pVogs with at least 3 per 1 kb [51], (4) greater than 3 kb with no BLASTn alignments to the NT database (January 2019) (e-value threshold: 1e–10), (5) BLASTn alignments to viral RefSeq database (v.89) (e-value threshold: 1e–10), and (6) less than 3 ribosomal proteins as predicted using the COG database [52].

### 2.9. Ethics

Faecal samples were collected from volunteers following acquisition of written consents according to study protocol APC055, approved by the Cork Research Ethics Committee (CREC).

### 2.10. Data Availability

Raw reads of both the 16S amplicon sequencing, and the virome WGS sequencing were uploaded to NCBI Sequence Read Archive (SRA) and are accessible under BioProject number PRJNA851256.

## 3. Results

We systematically tested changes in bacterial or viral composition in response to different short time (24 h) treatments. These treatments were: (1) phage addition (ΦAPCEc01 + FSI) vs. no addition (only FSI); (2) heat-killed phage + bacterial host addition (heat-killed ΦAPCEc01 + APC106 + FSI) vs. bacterial host addition (APC106 + FSI); (3) viable phage + bacterial host addition (viable ΦAPCEc01 + APC106 + FSI) vs. bacterial host addition (APC106 + FSI). The latter experiment was carried out twice, on separate occasions. In all iterations the fermenters were filled with sterile fastidious anaerobe broth medium, followed by addition of FSI (10% reactor volume) and bacterium APC106 (1% reactor volume, 1–5 × 10^8^ CFU) if needed. Phage ΦAPCEc01 (1% reactor volume, 1–5 × 10^9^ PFU) was introduced just before the second sampling (T1), two hours after the experimental start point to allow the system to stabilize, and to give time for the inoculated E. coli strain to acclimatise. Liquid samples were withdrawn before commencing the experiment (T0*–*0 h), every two hours as indicated (T1*–*2 h, T2*–*4 h, T3*–*6 h), and at the endpoint (T4*–*24 h). These conditions cover a wide range of treatments allowing an adequate comparison of the sophisticated faecal fermenters to the simple 50 mL centrifuge tube setup.

### 3.1. Treatment 1–Phage Addition vs. No Addition

Before the faecal fermentation was performed double-layer agar plates were prepared to test if the FSI had any phages that produce plaques on the *E. coli* APC106 lawn, used later for inoculation. We determined that there was no phage originating from the FSI that could lyse our model bacterium. Furthermore, double-layer agar plates were prepared throughout the study to verify the presence/absence of phage ΦAPCEc01. We recorded plaque formation only in the fermenters where the phage was externally added (Plastic tube 2–PT2, and Vessel 2–V2). However, during bioinformatic evaluation of the sequencing results, a contig similar to ΦAPCEc01 emerged in some unspiked samples (Treatment 1: Pt1 T0, Treatment 2: Pt1 T4,V1 T2 and T4, Treatment 3–fermentation 2: Pt1 T2, T3; V1 T2, T3; Treatment 3–fermentation 3: Pt1 T3, T4; V1 T2, T3, T4) with low abundance. We believe this is a similar bacteriophage that was assigned to the same taxonomic unit. The threshold for viral contig identification was kept in a range where the most number of them could be assigned with the least possible false positive identification.

**Figure 1:**
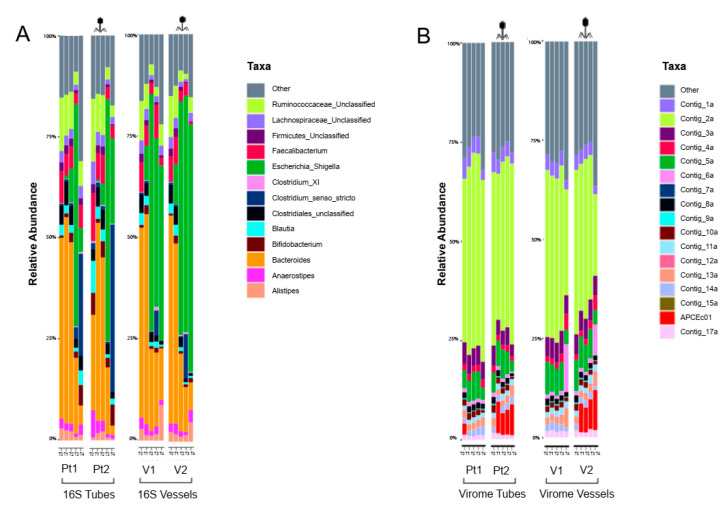
Sequencing results of faecal fermenter tubes and vessels during treatment 1 (no addition vs. ΦAPCEc01 addition, FSI batch1) (**A**) stacked bar plots showing different bacterial abundances on genus level (**B**) stacked bar plots showing different viral abundances.

First, we sought to examine the effect of adding a high-titer (10^9^ PFU) preparation of a previously isolated human faecal phage (ΦAPCEc01) into a faecal fermentation initiated with FSI to explore any possible off-target effects. Panel A in Figure 1 depicts the bacterial composition of the fermenters. Not surprisingly, the donor’s microbiota was overwhelmed with *Bacteroides* species, a common member of the human gut bacteriome [53]. Several other common genera like *Faecalibacterium*, *Ruminococcacea* unclassified Clostridiales and *Lachnospiraceae* were also present at lower abundance (5–10%), although the lack of detectable *Escherichia*/*Shigella* in the starting material at 0 h (T0) is noteworthy. During the course of the experiment, the bacterial composition changed rapidly. Expansion of the previously undetected *Echerichia*/*Shigella* started as early as 2 h (T1) after the start. This trend continued for time points T2 and T3 (4 and 6 h), mainly at the expense of the *Bacteroides* group. However, this outgrowth was slower in the case of the tubes, probably due to the absence of constant stirring. After 6 h, relative abundance of *Escherichia*/*Shigella* group reached around 50% in all four fermenters. This remained stable after 24 h (T4) in the vessels but decreased in the tubes where the *Clostridium* sensu stricto group took over around 25–30% total abundance.

Virome composition of the fermenter stayed stable throughout the 24 h experiment. The contig representing our externally added phage is marked with red (Figure 1B). It appeared at 2 h timepoint, right after introduction into the fermenter liquid, according to the study setup. The amount of ΦAPCEc01 stayed stable with around 10% abundance in the whole virome throughout the rest of the experiment.

We observed no significant difference between the treatment and the control (phage addition vs. no addition) nor between the fermenter setup (vessels vs. tubes) at the level of resolution provided by 16S rRNA analysis. Other important community measures (α and β diversities) also corroborate these findings (  Supplementary Figures S1, S2 and S4). Virome composition was also indistinguishable within the samples or between the fermenters, while the introduced faecal phage maintained constant abundance in the fermenter (Supplementary Figures S3 and S5).

### 3.2. Treatment 2–Heat-Killed Phage + Bacterium Addition vs. Bacterium Addition

During the next 24 h fermentation, a phage-sensitive bacterium, *E. coli* APC106, was introduced to supplement the donor’s faecal microbiota in both tubes and vessels (PT1, PT2, V1, V2). Since our 16S sequencing pipeline cannot discriminate between the inoculated and native *E. coli* strains, Chromocult selective agar was used (aerobic O/N growth at 37 °C) to follow the fate of the externally added bacterial strain. Faecal inoculum samples plated on Chromocult plates showed dark purple colonies exclusively. On the other hand, APC106 produced distinctive blue-coloured colonies that were easily distinguishable from the inherent *E. coli* strains. In addition, we continued monitoring the presence of phages capable of infecting APC106 in all fermenters with double-layer agar spot assays using samples withdrawn during the course of the fermentation. 1% O/N culture of APC106 bacterium (1–5 × 10^8^ *C*FU) and heat-killed ΦAPCEc01 (1–5 × 10^9^ PFU) stock preparation was added two hours apart to one vessel and tube, respectively (PT2, V2). The other was supplemented with viable bacterium only (PT1, V1), and served as a non-treated control. We applied heat treatment at 95 °C for 15 min to inactivate the phage stock. We confirmed the loss of infective capability on double-layer agar plates using APC106 in the overlay agar.

**Figure 2:**
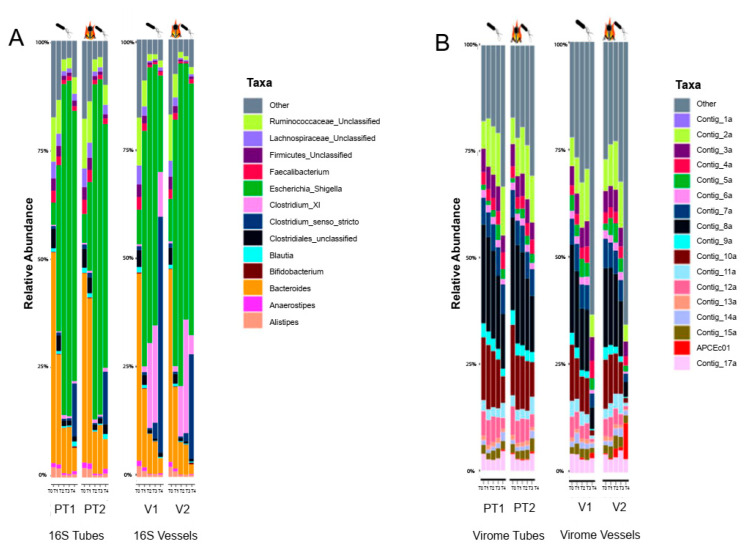
Sequencing results of faecal fermenter tubes and vessels during treatment 2 (*E. coli* APC106 addition vs. *E. coli* APC106 + heat-killed ΦAPCEc01 addition, FSI batch2) (**A**) stacked bar plots showing different bacterial abundances on genus level (**B**) stacked bar plots showing different viral abundances.

16S bacterial composition of the fermenters at T0 (0 h) was no different from the previous treatment setup (Figure 2A), except for the appearance of the externally added *E. coli* strain. The *Escherichia*/*Shigella* group represented around 10% abundance in all fermenters. As expected, the growth of this taxon was more pronounced reaching an overwhelming 60–80% overall abundance. However, expansion happened again more swiftly inside the vessels. Chromocult agar plating verified the presence of APC106 throughout the experiment. It is important to mention that the expansion of clostridia taxa (*Clostridium* XI and *Clostridium* sensu stricto) was only observed in vessels from samplings T2 and T3 (4 and 6 h). In V1, (only viable bacterium addition) *Clostridium* sensu stricto reached around 50% abundance after 24 h. A similar trend was visible in the tubes during the first treatment experiment. According to α and β diversity metrics we observed no significant difference in overall 16S bacterial composition between the control and treatment fermenters, and only a small but not significant deviation in the bacterial α and β diversities between vessels and tubes throughout the samplings.

The virome composition again showed high stability between samplings. Those contigs with less than 5% abundance (named “other”) showed a gradual increase in both tubes. Notably, the same fraction of contigs reached more than 50% abundance in the vessels after 24 h (T4). From the sequencing data, it is evident that the overall virome composition of the FSI is different from the previous fermentation. This is in contrast with the 16S distribution which did not show this level of deviation. The reason for this difference could well be that the FSI stock used to carry out the fermentation was prepared from a separate faecal donation from the same donor. Metadata from the donor was not available, so there is no information about changed lifestyle or medication.

Fermenters supplemented with the heat-killed phage showed no lytic activity against the sensitive host. The only exception was vessel 2 which received both heat-killed phage and *E. coli* APC106. Double layer agar plates showed no phage presence after 24 h of incubation. Close examination of this sample also showed a slight increase of the contig representing the introduced faecal phage (Figure 2B). A possible explanation for this is the survival of a small number of viruses in the heat-killed liquid below the detection limit of the spot assay that could have propagated above the detection limit by the end of the experiment.

Findings of treatment 2 experiments further corroborate our previous results indicating no significant difference between vessels and tubes, further strengthening the idea of using 50 mL centrifuge tubes as simple faecal fermenter devices (Supplementary Figures S1–S5).

### 3.3. Treatment 3–Viable Phage + Bacterium Addition vs. Bacterium Addition

In the subsequent fermentation, viable ΦAPCEc01 was added along with APC106 into one of the vessels/tubes (PT2 and V2) and compared to the non-treatment controls where only APC106 was added (PT1 and V1). We performed two 24-h-long fermentations with the same setup one week apart from each other. Due to the variation in virome composition across different batches of the FSI, we wanted to test both stocks used so far (FSI-A used for fermentation 2, and FSI-B used for fermentation 3). As before, Chromocult plates and double-layer agar spot assays were used to monitor the presence/absence of the introduced phage and bacterium along the fermentation.

**Figure 3:**
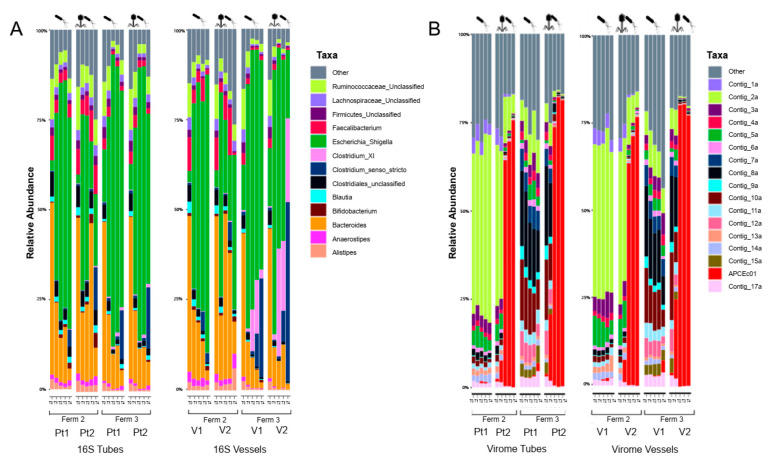
Sequencing results of faecal fermenter tubes and vessels during treatment 3 (*E. coli* APC106 addition vs. *E. coli* APC106 + viable ΦAPCEc01 addition; fermentation 2–FSI batch1, fermentation 3–FSI batch2) (**A**) stacked bar plots showing different bacterial abundances on genus level (**B**) stacked bar plots showing different viral abundances.

16S bacterial composition showed similar overall distributions as seen during treatment 1 and treatment 2 (Figure 3A). Fermentation 2 carried out with FSI-A showed an even expansion of *Escherichia*/*Shigella* group at T1 sampling across all fermenters with abundance reaching around 50–55%. At this point, we introduced the phage into PT2 and V2, respectively. This resulted in a marked drop in *Escherichia*/*Shigella* abundance within T2 and T3 (4 and 6 h) samples, followed by moderate recovery after 24 h (T4). Recovery was more pronounced in V2. The same trend was not recorded in the case of the non-treatment controls (PT1 and V1), where *Escherichia*/*Shigella* abundance stabilized at around 60–70% during T2–T4. Fermentation 3 using FSI-B showed slightly different dynamics. The abundance of *Escherichia*/*Shigella* taxon in the beginning is generally higher at T0 as seen before, that is probably due to the different FSI batch. This can also explain the enormous expansion in abundance (~60–80%) in the non-treated fermenter tube/vessel (PT1, V1). Viable phage addition after 2 h had a moderate effect on the *Escherichia*/*Shigella* group, but their proportion in the bacteriome remained below the abundance of the non-treated samples throughout the experiment. Similar to treatment 2, (Figure 2A, started with the same FSI) expansion of the same clostridia taxa was measured in the vessels. Interestingly this expansion was more evident in V2 where infective phage was present. Plating fractions of liquid samples on Chromocult plates revealed a decrease of APC106 only in the treated fermenter tube/vessel (PT2, V2) during both fermentations (2 and 3), indicating specific phage activity.

Virome sequencing results of fermentation 2 and 3 depict a clear-cut picture about different general compositions resulting from the different FSIs used, as seen before in treatment 1 and treatment 2 (Figure 1B and Figure 2B). However, more evident is the difference between the treated and non-treated tubes/vessels (Figure 3B). The contig representing ΦAPCEc01 appears at time point T1, after introduction into both PT2 and V2. Propagation of the externally added faecal phage is beyond doubt, since the representation of this contig climbs swiftly to around 60–70% of the overall viral reads, and stabilizes around 75–80% for the remainder of both fermentation 2 and 3. This was accompanied by a marked increase in the infective titre of the externally added faecal phage (ΦAPCEc01) inside the treatment vessel/tube as reported by double-layer agar plate spot assays. This could only be achieved by propagation inside the fermenters through specific killing of the bacterial host.

16S sequencing results again showed no significant differences between the treatment and the control fermenters (Figure 3A) and no statistically significant difference was observed between the vessels and the tubes (Supplementary Materials). Despite the efficient propagation of the externally added phage, the overall difference between the treatment and control fermenters was also moderate as reported by Jaccard distances. More importantly, we found no significant difference between the datasets originating from tubes and vessels.

## 4. Discussion

Bacterial viruses can find and lyse their hosts in different environments [54]. Using phages in human medicine against pathogenic bacteria was overtaken by the discovery of powerful antimicrobial agents, antibiotics. These drugs saved countless lives over the last century but their overuse gave rise to multidrug-resistant human pathogenic strains. These “superbugs” pose a real threat to modern medicine [55,56]. Phages are promising alternatives to antibiotics however, their efficacy and safety assessment requires rigorous in vivo clinical testing [57]. This is usually preceded by in vitro data collection (e.g., faecal fermentations) for scientific and economic reasons [58]. Our concept relied on using simple screw-cup 50 mL centrifuge tubes in parallel with a standardised faecal fermentation system allowing us to assess them both as surrogates for the complex human gut. A relatively short, 24 h long timespan was selected to reflect the average transit time in the human colon.

We conducted a series of in vitro fermentations using two different setups following the introduction of a human faecal standard inoculum produced from the faecal sample of an anonymous donor. On one hand, we used a sophisticated two-vessel fermentation unit specially designed to conduct microbial (or faecal) fermentations, while on the other hand, we used a more simplified model involving plastic centrifuge tubes as fermenters. Both systems were run for 24 h given the plastic tube method required fermentations to run in a batch mode. This short-term experiment is a simple gut model and of course it is unable to capture all changes that might occur within a true gut microbiome. However, we show that the addition of a single faecal phage did not significantly change the microbial content of either system during this period. We also demonstrated that even in the presence of their host, heat-killed phage did not have any significant effect on the bacterial or viral composition of the two systems. In contrast, we observed massive propagation of the phage when its bacterial host was also introduced into the fermenter, proving the capability of ΦAPCEc01 to actively lyse its hosts in both models of a gut environment despite the short fermentation time. Bioinformatic analysis of the sequencing data showed that the donor bacterial composition does not change significantly as a result of the different 24 h-long treatment conditions (only phage addition vs. no addition; heat killed phage + bacterium addition vs. bacterium addition; viable phage + bacterium addition vs. bacterium addition). Parallel investigation targeting the viral fraction has corroborated the same lack of global effect in this timeframe. We observed no significant change in viral composition except for the massive propagation of the external phage in the presence of its host. We also noted an altered baseline viral composition in two different faecal donations by the same individual. It is hard to pinpoint the reasons behind this phenomenon without detailed metadata about the donor (change in diet, antibiotic or other prescription drug intake). Nevertheless, these results proved the feasibility of both model fermentation systems in the investigated timeline. Furthermore, we verified that an externally added faecal phage could effectively lyse its host in this artificial environment and propagate to a high titre.

From the two different systems employed here, the 50 mL centrifuge tube system outperformed the more sophisticated faecal fermentation vessels in some metrics (cost; hands-on time to set up and run). Employing this simple technique, we can avoid investment costs to buy expensive equipment, but we can also generate less volume of biohazardous waste, which leads towards a reduced carbon footprint. However, we also need to mention that neither our closed vessel, nor the plastic tube systems can be run on a continuous manner. Meaning, if a longer period is to be investigated faecal fermentation vessels are still inevitable. Based on the results we conclude that using plastic centrifuge tubes is a serviceable system to model aspects of the gut environment when 24 h-long incubation periods are suitable. In addition, our observation of the temporal instability of the individual phageome without marked differences in 16S rRNA composition can be important for future gut virome studies.

We believe these results demonstrate the highly specific nature of phages residing in the human gut. Our model environment experimentally proved the ability of a phage of human gut origin to find and effectively lyse its host without effects on other bacteria. This underlines the effectivity and most of all selectivity of phages even in this complex environment. Our results also have implications for the safety of phage therapy, since possible off-target effects on the bacteriome could hinder the use of phages as therapeutic agents. Contrary to commonly used antibiotics that have broad range effects on numerous indigenous beneficial bacteria in the human gut, short-term use of a phage did not cause disruptions to microbiota composition. Of course, more robust studies are warranted to investigate any long-term effect phage might have on the inherent microbiota. Ideally, Illumina 16S amplicon sequencing should be replaced in the future by another platform that is capable of higher taxonomical resolution (like MinION from Oxford Nanopore technologies) [59,60]. Nevertheless, according to current standards in human gut microbiome studies, this paper supports the use of plastic centrifuge tubes as surrogate faecal fermenters for short-term fermentations and also underlines the efficacy and safety of using phages as specific antimicrobials / therapeutic agents during targeted clinical trials.

## 5. Conclusions

Phage therapy is undoubtedly gaining momentum worldwide resulting in the need for supportive in vitro research data. Commonly applied models of the human colon are sophisticated yet cumbersome to operate, and often produce large amounts of biohazard waste. In this study, a commercially available two-vessel fermentation system was compared with using 50 mL plastic screw-cap vials as alternatives in a short-term (24 h) setup. Applying a wide range of treatments, we demonstrated that our simple setup is interchangeable with the complex faecal fermentation system in this timespan. No statistical differences were recorded when comparing the bacterial or viral compositions of these systems. We determined that a lytic phage previously isolated from a human faecal sample can lyse its host and propagate to a high titre when introduced into this complex environment. Additionally, we have shown the highly specific nature of host depletion, without any off-target effect detectable during the 24 h duration of the experiment.

## Data Availability

The data presented in this study are openly available in NSCI Sequence read archive (SRA) at reference number PRJNA851256.

## Supplementary Materials

The following are available online at https://www.mdpi.com/article/10.3390/v14122632/s1, Figure S1: Alpha diversity metrics across all fermentations, Figure S2: Weighted Unifrac PCoA graph of 16S samples separated by fermentations, Figure S3: Jaccard PCoA graph of virome samples separated by fermentations, Figure S4: Weighted Unifrac PCoA graph of all 16S samples, Figure S5: Jaccard PCoA graph of all virome samples.
