# Supplementary material for: Comparing In Vitro Faecal Fermentation Methods as Surrogates for Phage Therapy Application
Source: Viruses. 2022 Nov 25;14(12):2632. doi: 10.3390/v14122632 (PMC9786711; doi:10.3390/v14122632)
Supplement: Supplementary file 1 [file viruses-14-02632-s001.zip › Supplementary Figure legends.pdf]

Supplementary Figure legends

- Supplementary Figure S1.: Box plots showing Simpson's diversity index across control and treatment 16S samples throughout all fermentations. Fermentation 1 - no addition vs. ΦAPCEc01 addition (FSI batch1); Fermentation 2 - *E. coli* APC106 addition vs. *E. coli* APC106 + viable ΦAPCEc01 addition (FSI batch1); Fermentation 3 - *E. coli* APC106 addition vs. *E. coli* APC106 + viable ΦAPCEc01 addition (FSI batch2); Fermentation 4 - *E. coli* APC106 addition vs. *E. coli* APC106 + heat-killed ΦAPCEc01 addition, FSI batch2)

- Supplementary Figure S2.: PCoA graph showing weighted unifrac differences of 16S samples according to each fermentation: Fermentation 1 - no addition vs. ΦAPCEc01 addition (FSI batch1); Fermentation 2 - *E. coli* APC106 addition vs. *E. coli* APC106 + viable ΦAPCEc01 addition (FSI batch1); Fermentation 3 - *E. coli* APC106 addition vs. *E. coli* APC106 + viable ΦAPCEc01 addition (FSI batch2); Fermentation 4 - *E. coli* APC106 addition vs. *E. coli* APC106 + heat-killed ΦAPCEc01 addition, FSI batch2). Sampling times: T0-0h, T1-2h, T2-4h, T3-6h, T4-24h

- Supplementary Figure S3.: PCoA graph showing Jaccard indexes of virome samples according to each fermentation: Fermentation 1 - no addition vs. ΦAPCEc01 addition (FSI batch1); Fermentation 2 - *E. coli* APC106 addition vs. *E. coli* APC106 + viable ΦAPCEc01 addition (FSI batch1); Fermentation 3 - *E. coli* APC106 addition vs. *E. coli* APC106 + viable ΦAPCEc01 addition (FSI batch2); Fermentation 4 - *E. coli* APC106 addition vs. *E. coli* APC106 + heat-killed ΦAPCEc01 addition, FSI batch2). Sampling times: T0-0h, T1-2h, T2-4h, T3-6h, T4-24h

- Supplementary Figure S4.: PCoA graph showing weighted unifrac differences of all 16S samples

- Supplementary Figure S5.: PCoA graph showing Jaccard indexes of all virome samples
