# Supplementary figures and images for: Comparing In Vitro Faecal Fermentation Methods as Surrogates for Phage Therapy Application
Source: Viruses. 2022 Nov 25;14(12):2632. doi: 10.3390/v14122632 (PMC9786711; doi:10.3390/v14122632)

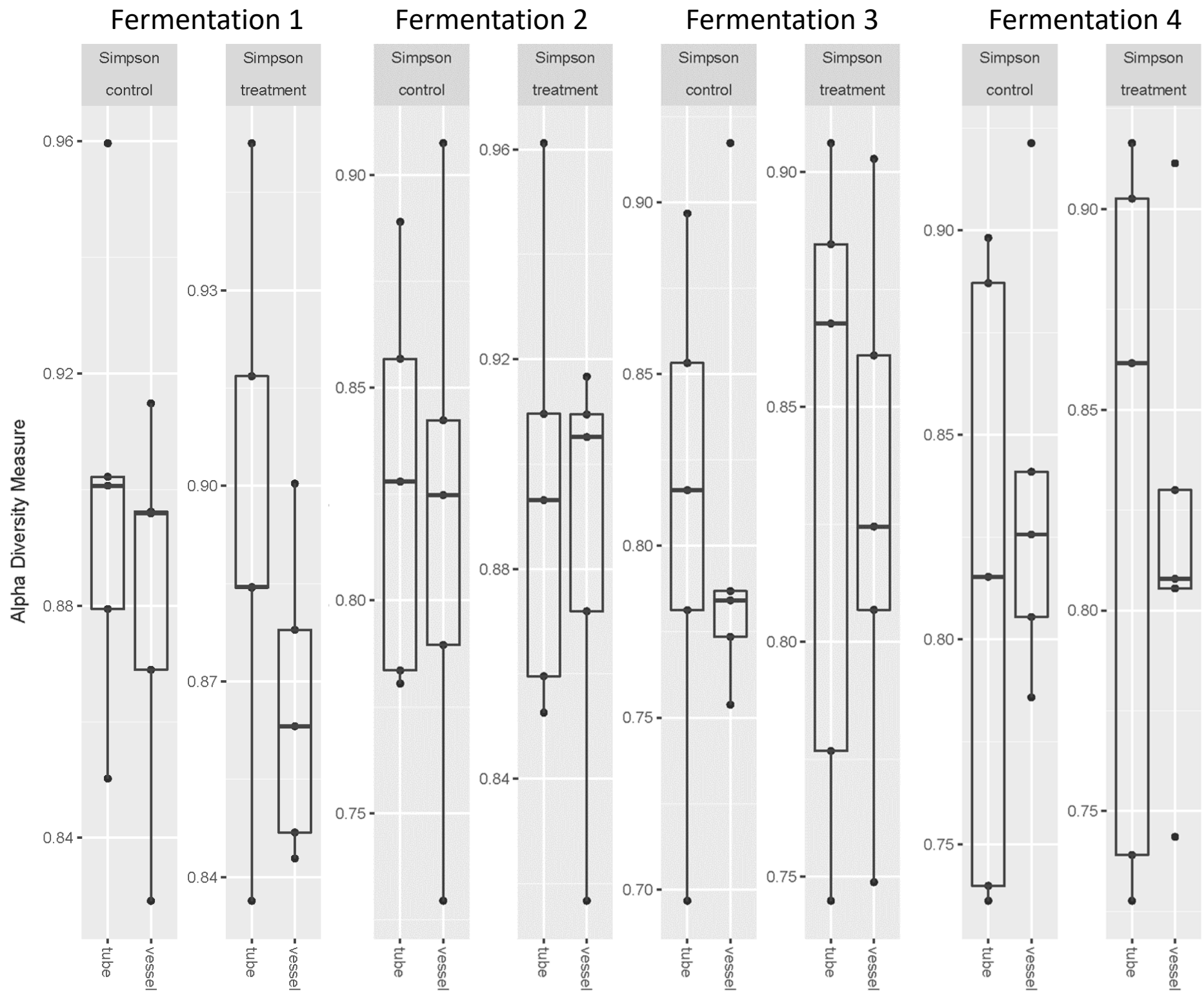

Supplement: Supplementary file 1 [file viruses-14-02632-s001.zip › S1_16S alphadiversity figure.pdf]

# PCoA – Unifrac Weighted

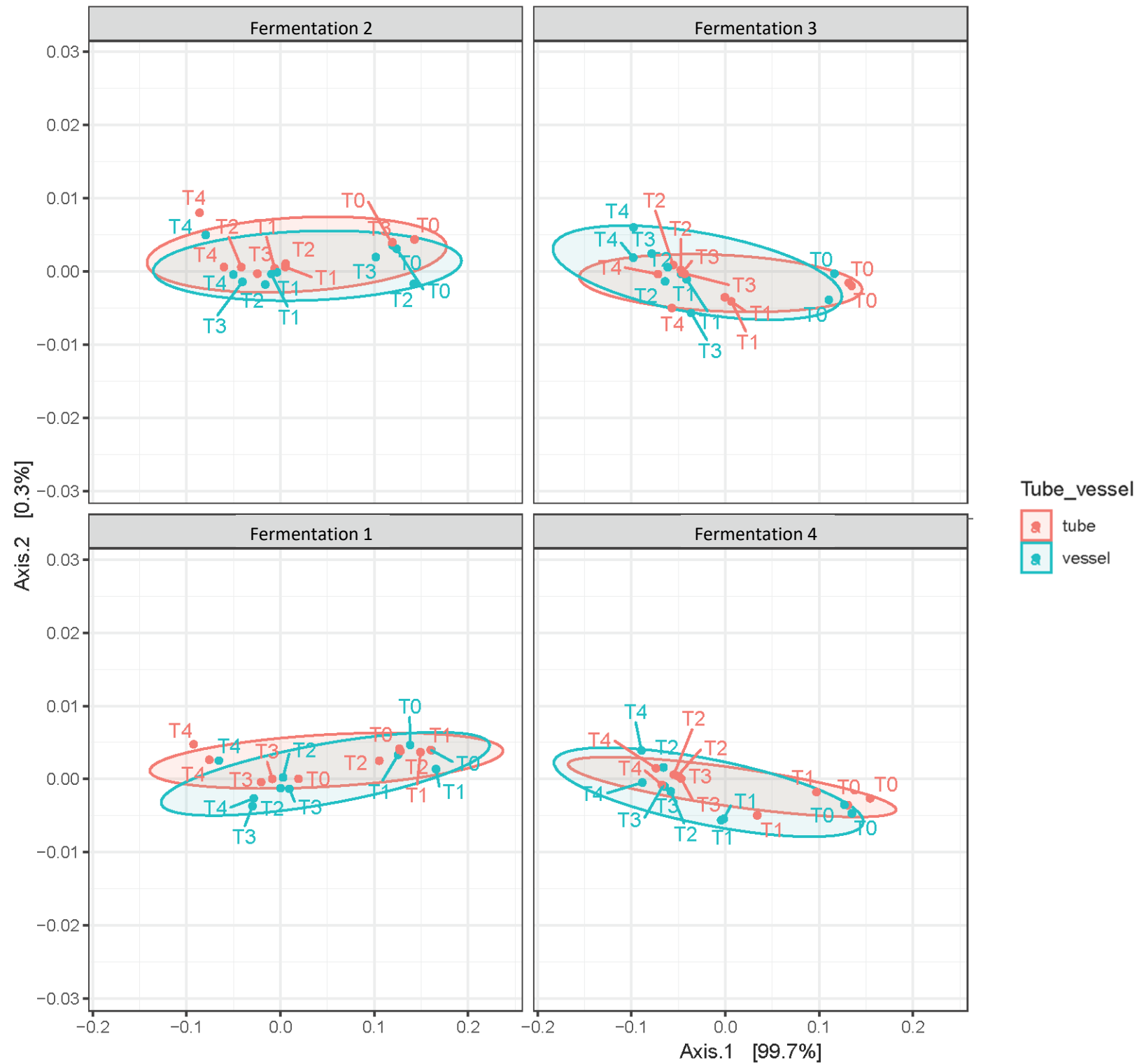

Supplement: Supplementary file 1 [file viruses-14-02632-s001.zip › S2_16S_PCoA_w_time_labels figure.pdf]

PCoA – Jaccard

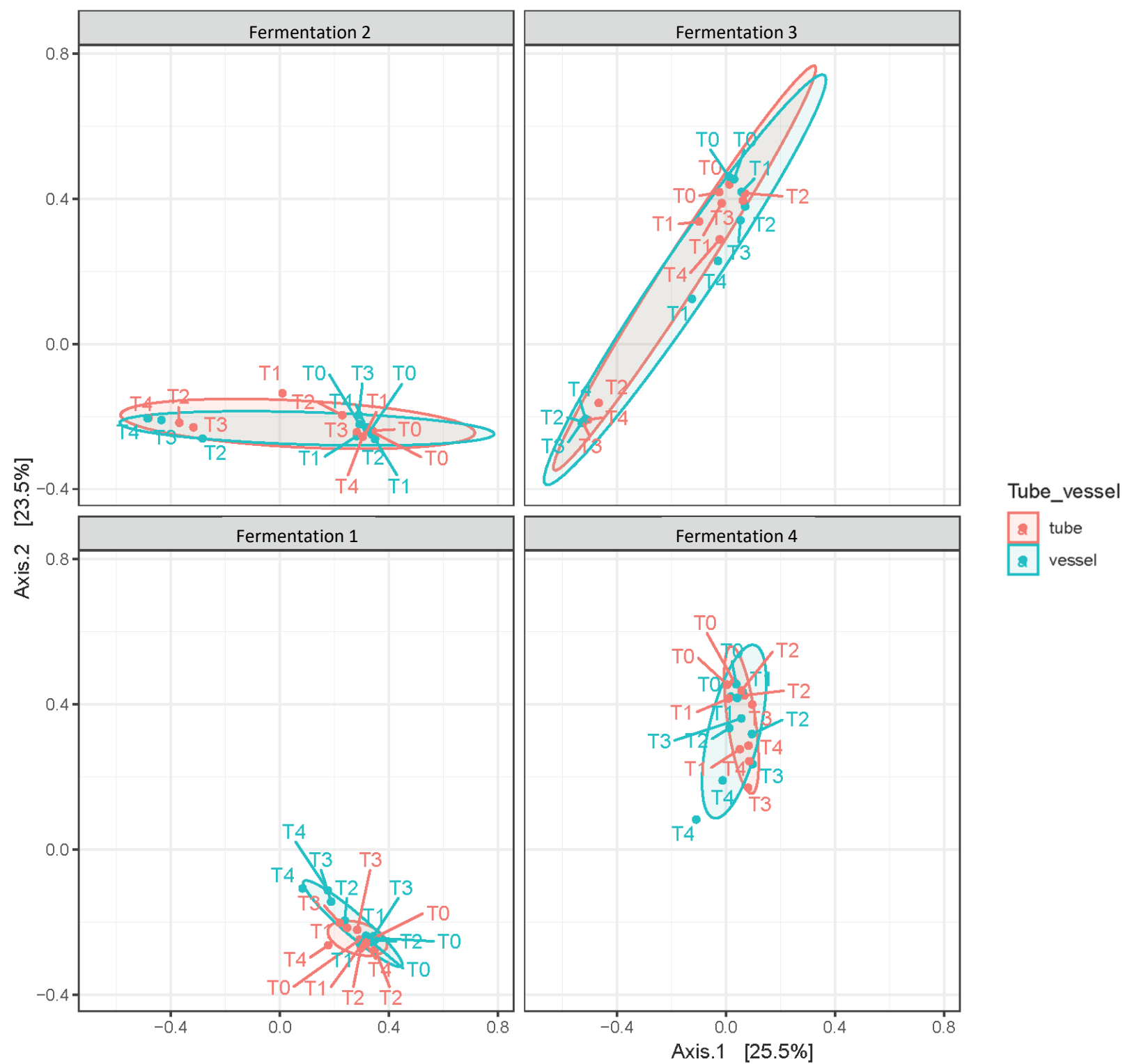

Supplement: Supplementary file 1 [file viruses-14-02632-s001.zip › S3_Virome_PCoA_w_time_labels figure.pdf]

PCoA - Weighted Unifrac

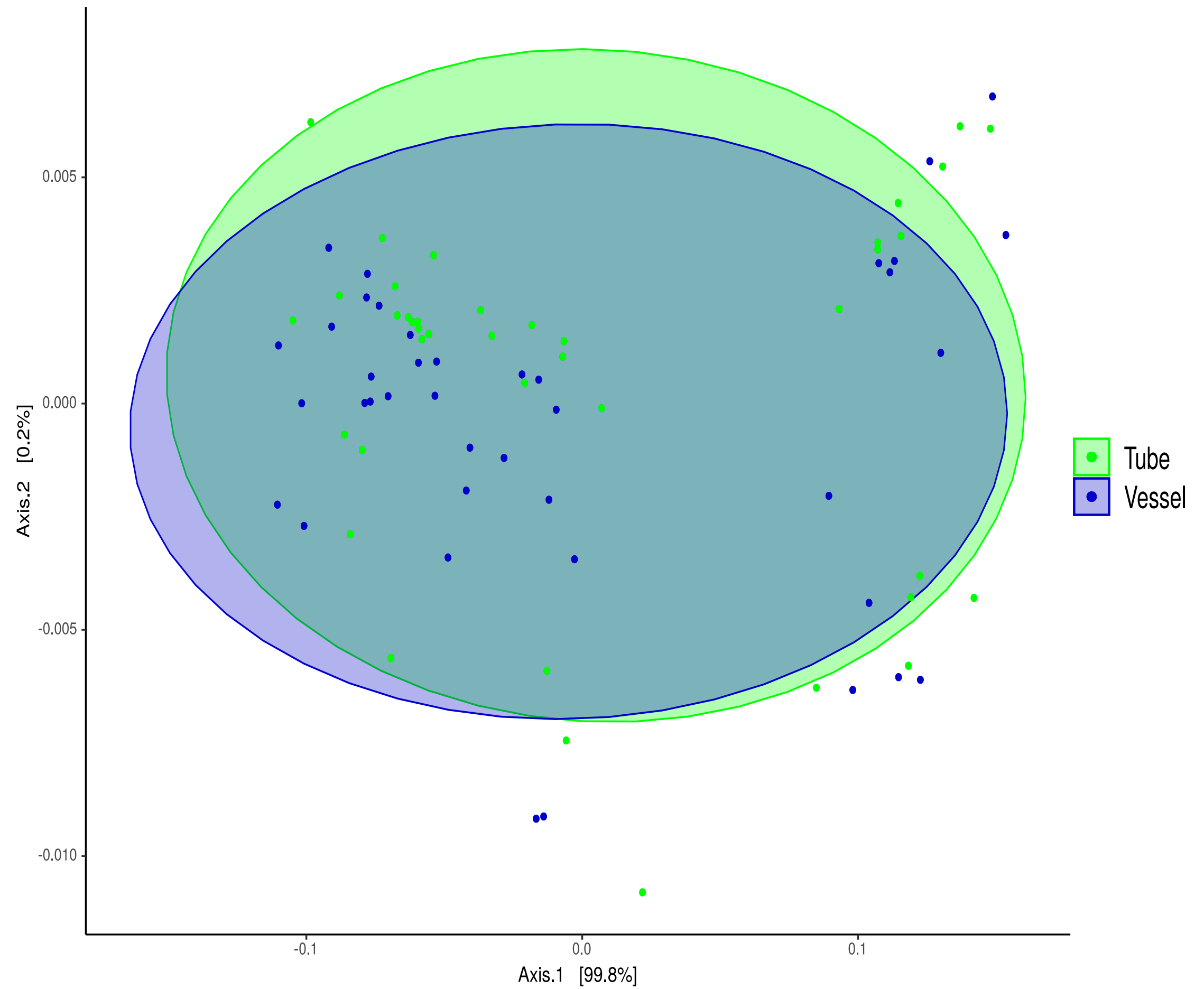

Supplement: Supplementary file 1 [file viruses-14-02632-s001.zip › S4_TV_16S.pdf]

PCoA - Jaccard

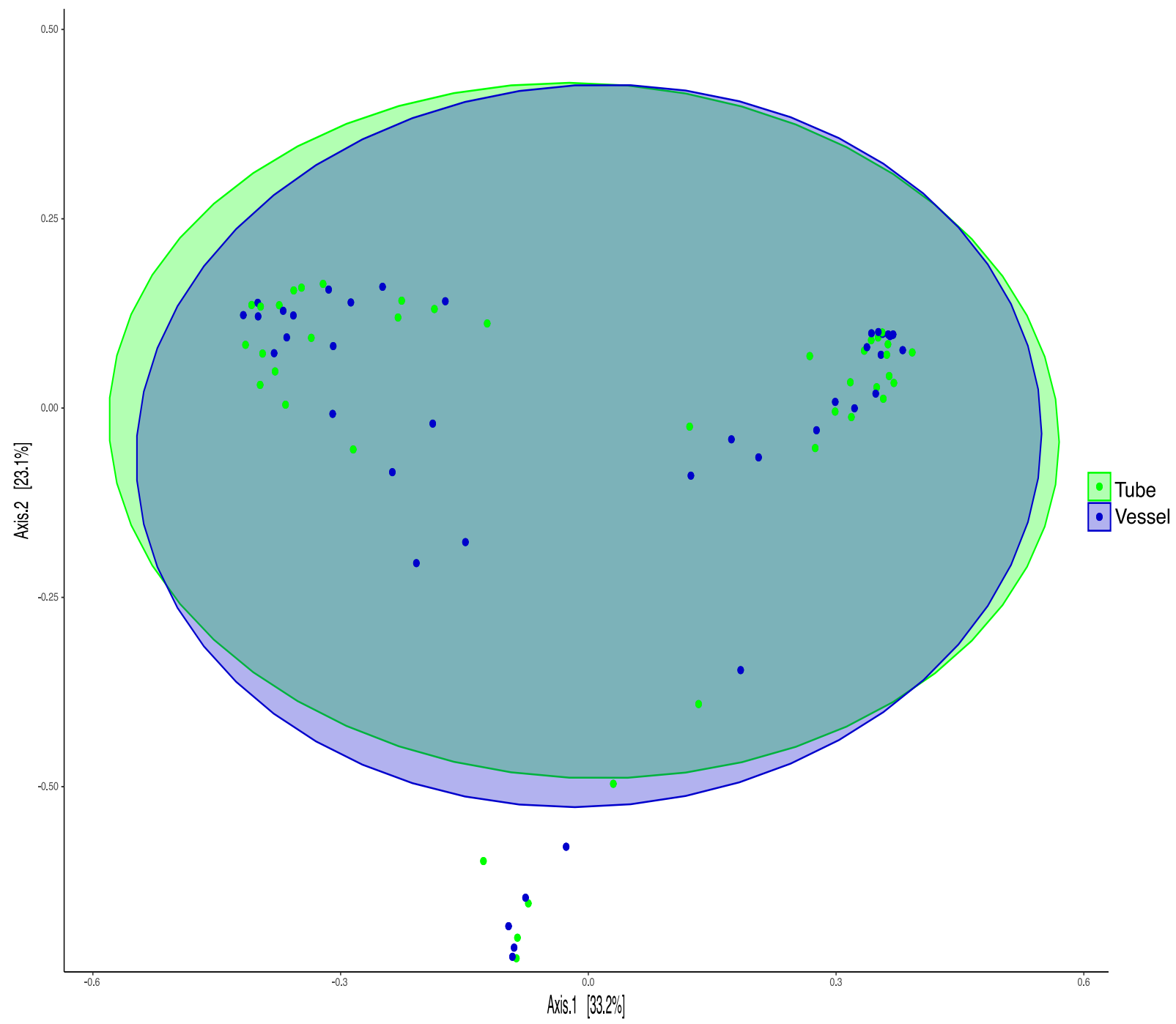

Supplement: Supplementary file 1 [file viruses-14-02632-s001.zip › S5_TV_virome.pdf]
